# Explainable AI to Facilitate Understanding of Neural Network-Based Metabolite Profiling Using NMR Spectroscopy

**DOI:** 10.3390/metabo14060332

**Published:** 2024-06-14

**Authors:** Hayden Johnson, Aaryani Tipirneni-Sajja

**Affiliations:** Magnetic Resonance Imaging and Spectroscopy Lab, Department of Biomedical Engineering, The University of Memphis, Memphis, TN 38152, USA; htjhnson@memphis.edu

**Keywords:** explainable AI, neural networks, NMR metabolomics, NMR spectroscopy, integrated gradients, metabolite profiling, machine learning

## Abstract

Neural networks (NNs) are emerging as a rapid and scalable method for quantifying metabolites directly from nuclear magnetic resonance (NMR) spectra, but the nonlinear nature of NNs precludes understanding of how a model makes predictions. This study implements an explainable artificial intelligence algorithm called integrated gradients (IG) to elucidate which regions of input spectra are the most important for the quantification of specific analytes. The approach is first validated in simulated mixture spectra of eight aqueous metabolites and then investigated in experimentally acquired lipid spectra of a reference standard mixture and a murine hepatic extract. The IG method revealed that, like a human spectroscopist, NNs recognize and quantify analytes based on an analyte’s respective resonance line-shapes, amplitudes, and frequencies. NNs can compensate for peak overlap and prioritize specific resonances most important for concentration determination. Further, we show how modifying a NN training dataset can affect how a model makes decisions, and we provide examples of how this approach can be used to de-bug issues with model performance. Overall, results show that the IG technique facilitates a visual and quantitative understanding of how model inputs relate to model outputs, potentially making NNs a more attractive option for targeted and automated NMR-based metabolomics.

## 1. Introduction

Nuclear magnetic resonance (NMR) spectroscopy is a non-destructive, highly reproducible, quantitative analytical technique used primarily for identifying molecules based on their atomic structure [1]. NMR spectroscopy is also one of the major techniques used in complex mixture analysis for applications such as metabolomics, reaction monitoring, food analysis, and more [1,2,3,4]. Traditionally, the manual quantification of analytes in NMR spectra has been a tedious, slow, user-dependent process requiring a trained spectroscopist with domain knowledge of the analytes. Semi-automated and automated software have been developed for metabolite profiling from NMR data (e.g., Chenomx, Magmet, and LipSpin) [5,6]; however, these techniques are slower and less scalable compared to processing using neural networks (NNs). Despite past approaches described in the literature [7,8] and the current surge of interest in artificial intelligence (AI), NNs have yet to gain traction in the realm of targeted NMR profiling. The black box nature of NNs may hinder widespread acceptance. In this work, we aim to make neural networks a more attractive option for NMR analyte profiling by proposing the use of explainable AI (XAI) to address the issue of model understandability.

NNs have proven exceptional across many tasks and domains; however, the many layers and nonlinear transformations of a NN make understanding how a model reaches a decision essentially impossible by simply inspecting model outputs and parameters [9]. This lack of model understanding undermines user trust in the model and may mask underlying bugs or bias, discouraging use in critical applications. The black box nature of NNs has driven the emergence of the burgeoning field of XAI, which aims to facilitate an understanding of how AI models make predictions. Despite the extensive use of regression in AI, most of the work and research in XAI has pertained to classification problems, primarily in computer vision applications [10]. One major subset of XAI, attribution methods, aim to compute scores for each input feature, representing the contribution of that feature to the model’s output [11]. In this study, we implement the attribution-based integrated gradients (IG) algorithm [12] as an XAI approach to relate model inputs (NMR spectra of analyte mixtures) to outputs (predicted analyte concentrations). To the best of our knowledge, this is the first time XAI has been applied to NMR spectroscopic data.

The challenge involved in determining what influences a model’s decisions is a drawback to the NN approach to NMR-based analyte quantification. This work addresses this issue by applying a post hoc algorithm facilitating model interpretation—first in simulated mixture spectra of aqueous metabolites and then in experimentally acquired spectra of a complex lipid standard mixture and a hepatic lipid extract. This study explores and validates the IG method for improving the understanding of NN-based processing for NMR metabolite profiling.

## 2. Materials and Methods

### 2.1. Data Generation and Neural Network Training

**Figure 1:**
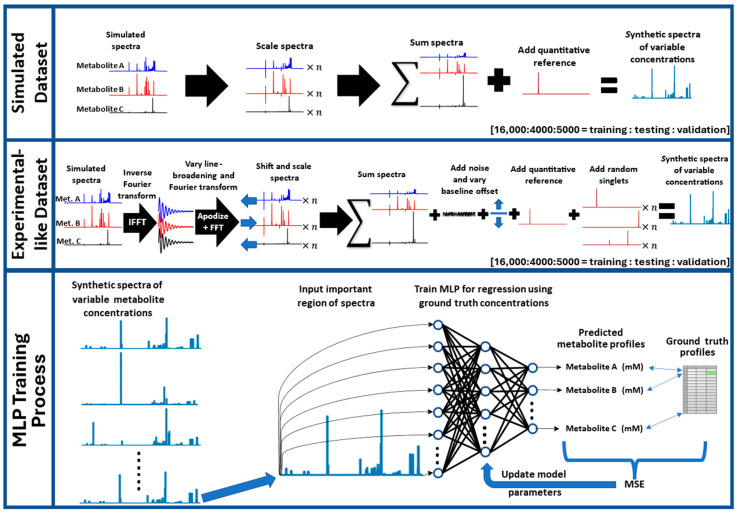
Data generation and neural network training workflows. The top two panels describe the datasets used in model training and testing, while the bottom panel provides an overview of the MLP training process. Abbreviations: MLP = multi-layered perceptron, MSE = mean squared error, IFFT = inverse fast Fourier transform, FFT = fast Fourier transform.

Simulated 400-MHz ^1^H-NMR spectra of common aqueous metabolites (taurine, choline, creatine, lactic acid, niacinamide, L-alanine, L-valine, maleic acid, and acetic acid) were downloaded from the human metabolome database (HMDB.ca). Subsequent processing steps were performed in Python (version 3.11.5) using the PyTorch (version 2.2.1) and NumPy (version 1.24.3) libraries for matrix operations and the nmrglue library (version 0.10) for processing NMR data (reading data, apodization, peak shifts, and Fourier transformations). The workflow for generating spectra for model training, testing, and validation is shown in Figure 1. Simulated spectra were individually scaled and summed to produce simulated mixture spectra. Eight metabolites (taurine, choline, creatine, lactic acid, niacinamide, alanine, and valine) were considered analytes and were scaled randomly to concentrations ranging uniformly from 1–50 mM, and maleic acid was added at 13.3 mM in each spectrum as a quantitative reference signal. This was repeated 10,000 times using all 8 metabolites in every spectrum and 10,000 times where each metabolite had a 50% chance of being left out to generate a total of 20,000 spectra which were split 16,000:4000 for training and testing, respectively. A further 5000-spectra validation dataset was generated using all 8 metabolites. Throughout this manuscript, this first set of training, testing, and validation data is referred to as the ‘Simulated Dataset’. A second dataset was generated using the same workflow but incorporating data augmentation mimicking realistic potential experimental variations including line-broadening (0.1–1.0 Hz), adding normally distributed noise (mean of zero and maximum standard deviation of ~1.3% of maleic acid peak height), shifting metabolite spectra (0–3.4 ppb for all resonances of a given analyte), shifting the baseline (up to ~7.5% of the maleic acid peak height), and adding up to three randomly scaled singlets at random chemical shifts (using the simulated acetic acid signal as a generic singlet). The second generated dataset is referred to as the ‘Experimental-Like Dataset’.

Synthetic spectra with specified compositions were generated in the same manner as the Simulated Dataset for validating XAI methods, including all eight analytes at 25 mM, 5 mM, and 1 mM, and all eight analytes at 25 mM plus two singlets added at randomly selected chemical shifts and amplitudes. To compare XAI results on models trained using either the Simulated or Experimental-Like Dataset, an additional spectrum with two added singlets was generated with noise added at the mean magnitude seen in training. A further spectrum was generated with only six metabolites (valine, alanine, creatine, choline, lactic acid, and niacinamide) at 25 mM each plus noise, which was generated to assist in comparisons between similar spectra with and without peak overlap.

Multi-layered perceptron (MLP) networks were developed with an input layer of 39,500 nodes (corresponding to 39,500 datapoints containing the relevant metabolite signal region [0.48 to 9.52 ppm]), a 200-node hidden layer, and an output layer of eight nodes (corresponding to the eight analyte concentrations). Before model training, the intensity values of the synthetic spectra in both training and testing datasets were normalized by dividing the intensity at each data point by the overall maximum intensity value achieved in the training/testing data. MLPs were trained using the Simulated Dataset and Experimental-like Dataset and are referred to as MLP-Sim and MLP-Exp, respectively, throughout the rest of this article. The mean squared error (MSE) between predictions and ground-truth concentrations was used as the loss function and the Adam optimizer was selected for optimization. A batch size of 128 was used and models were trained until loss converged. MLPs were developed using PyTorch. All computations were performed using a Quadro RTX 6000 (Nvidia, Santa Clara, CA, USA) and two Xeon Silver 4216s (Intel, Santa Clara, CA, USA).

### 2.2. XAI Implementation and Validation

The IG method is a post hoc analytical method for XAI compatible with differentiable machine learning models like NNs. The Captum library (version 0.7.0) was accessed for the IG algorithm, which was applied to MLP-Sim and MLP-Exp. The IG algorithm approximates the integral of the gradients of a model’s output with respect to its input [12]. IG requires the use of a baseline input, which is a neutral input used as a reference point when computing feature importance and represents the absence of features contributing to specific outputs (i.e., the baseline for alanine quantification should represent an alanine concentration of 0 mM). Therefore, a baseline was generated and utilized consisting of only the quantitative reference maleic acid at 13.3 mM (without noise added for MLP-Sim, and with noise added at the mean value used in data generation for MLP-Exp). Using the IG method, numbers termed attribution scores are computed for each input on a per-feature basis based on each feature’s contribution to the model’s prediction (i.e., we gain insight into how each datapoint in an input spectrum contributes to model-estimated concentrations).

Model predictions and attribution scores were evaluated on representative synthetic input spectra. Attribution scores were first calculated for three simple examples, with all eight metabolites present at equal concentrations (1, 5, and 25 mM). To examine how modification of the training dataset may affect attributions, we examined both MLP-Sim and MLP-Exp attribution scores for an input signal of all eight analytes at 25 mM plus two singlets randomly added near analyte resonances. XAI results were evaluated visually by comparing attribution scores to their respective input spectra and ground truth metabolite signals and assessing what was important for quantification (i.e., do the signals the algorithm determines as most important agree with the preferred resonances selected by spectroscopists for analyte quantification [5,13]). The XAI approach was additionally assessed quantitatively by summing the whole range as well as specific regions of interest (ROIs) of the attribution scores to determine if attribution score magnitude directly corresponds to analyte concentration.

### 2.3. XAI in Experimentally Acquired Lipid Spectra

**Figure 2:**
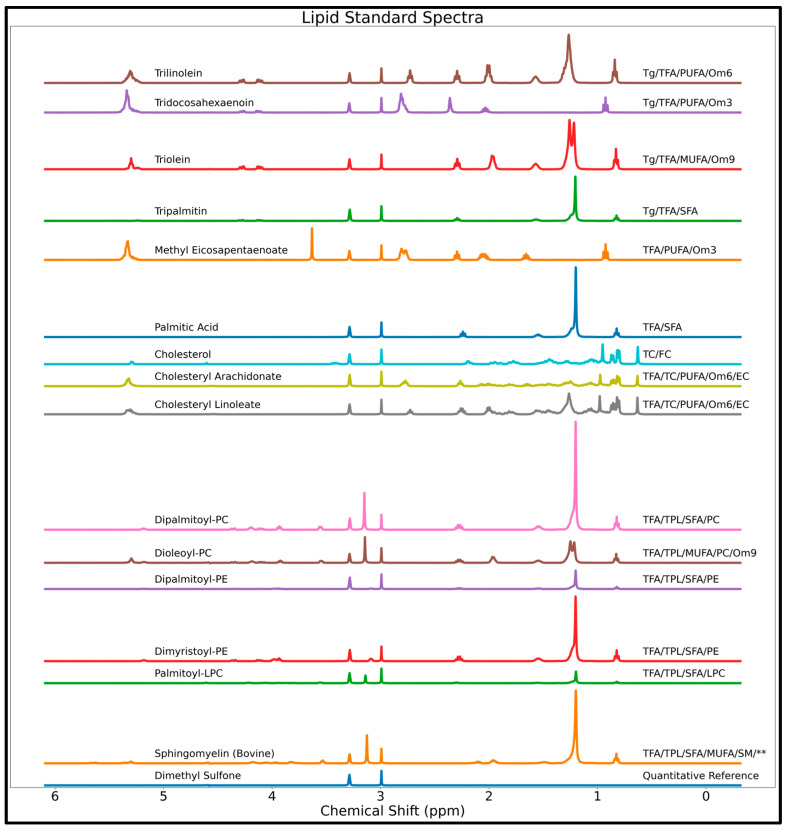
NMR spectra of 15 reference lipid standards used to train the MLP for lipid quantification, with the name of the standard listed on the left and the lipid groups quantified on the right. The ‘**’ symbol indicates that there are small amounts of unknown lipids in the SM. Abbreviations: DHA = docosahexaenoic acid; EC = esterified cholesterol; FC = free cholesterol; LA = linoleic acid; LPC = lysophosphatidylcholine; MUFA = monounsaturated fatty acids; Om3 = omega-3 fatty acids; Om6 = omega-6 fatty acids; Om9 = omega-9 fatty acids; PC = phosphatidylcholine; PE = phosphatidylethanolamine; PUFA = polyunsaturated fatty acids; SFA = saturated fatty acids; SM = sphingomyelin; TC = total cholesterol; TFA = total fatty acids; Tg = total triglycerides; TPL = total phospholipids.

After validating the IG approach for NN-based metabolite profiling in simulated spectra of aqueous metabolite mixtures, we explored the use of this XAI method in experimentally acquired spectra of complex lipid mixtures. Fifteen lipid reference standards from Nu-Chek Prep. (Elysian, MN, USA): tridocosahexaenoin [TriDHA], trilinolein, triolein, tripalmitin, cholesterol, methyl eicosapentaenoate [mEPA], palmitic acid, cholesteryl linoleate, and cholesteryl arachidonate, Tokyo Chemical Industry Co., (Tokyo, Japan):1,2-dipalmitoyl-sn-glycero-3-phosphocholine, 1,2-dioleoyl-sn-glycero-3-phosphocholine, 1,2-dipalmitoyl-sn-glycero-3-phosphoethanolamine, 1,2-dimyristoyl-sn-glycero-3-phosphoethanolamine, Avanti Polar Lipids, Inc. (Alabaster, AL, USA): 1-palmitoyl-2-hydroxy-sn-glycero-3-phosphocholine, and Matreya LLC (State College, PA, USA): sphingomyelin [bovine]). The standards were weighed individually to prepare 30 NMR samples (2 samples per standard) at various concentrations, and ^1^H-NMR scans of these 30 samples obtained using a 400-MHz JEOL ECZ spectrometer (JEOL Ltd., Tokyo, Japan) were used to train an MLP for lipid quantification similar to MLP-Exp above (i.e., Adam optimizer, 200-node hidden layer, MSE loss function, and using a training dataset utilizing experimental variations and additional non-analyte signals [tetramethylsilane, water, and random singlets]) [14]. A spectrum for each lipid reference standard is shown in Figure 2. Eighteen lipid parameters were quantified and thus the final output layer consisted of 18 nodes (corresponding to total triglycerides [Tg], total cholesterol [TC], total phospholipids [TPL], total fatty acids [TFA], polyunsaturated fatty acids [PUFA], monounsaturated fatty acids [MUFA], saturated fatty acids [SFA], linoleic acid [LA], docosahexaenoic acid [DHA], phosphatidylcholine [PC], lysophosphatidylcholine [LPC], phosphatidylethanolamine [PE], sphingomyelin [SM], omega-3 fatty acids [Om3], omega-6 fatty acids [Om6], omega-9 fatty acids [Om9], free cholesterol [FC], and esterified cholesterol [EC]).

A mixture spectrum containing 13 lipid standards (all standards used in training except 1-palmitoyl-2-hydroxy-sn-glycero-3-phosphocholine and SM) was evaluated with the IG algorithm using a baseline spectrum of only the quantitative reference substance (dimethyl sulfone [DMSO2]) and NMR solvent (deuterated chloroform, deuterated methanol, and deuterium oxide—16:7:1—*v*/*v*/*v*). A spectrum of hepatic lipids extracted from a Nile Grass rat obtained in a prior study [15] was similarly assessed with the IG algorithm. A more detailed description of the sample preparation, data acquisition, and model training can be found in the previous manuscript, which validated this lipid-profiling MLP model [14] (MLP-3 is the specific model and Mix 2 is the specific lipid mixture used for this study). Attribution results for lipids are assessed primarily visually, with peaks deemed important by the IG method compared both to lipid standard signals (Figure 2) and to resonances used by human spectroscopists for lipid quantification (Supplementary Figure S1) [5,15].

## 3. Results

### 3.1. XAI with Simulated Aqueous Spectra

**Figure 3:**
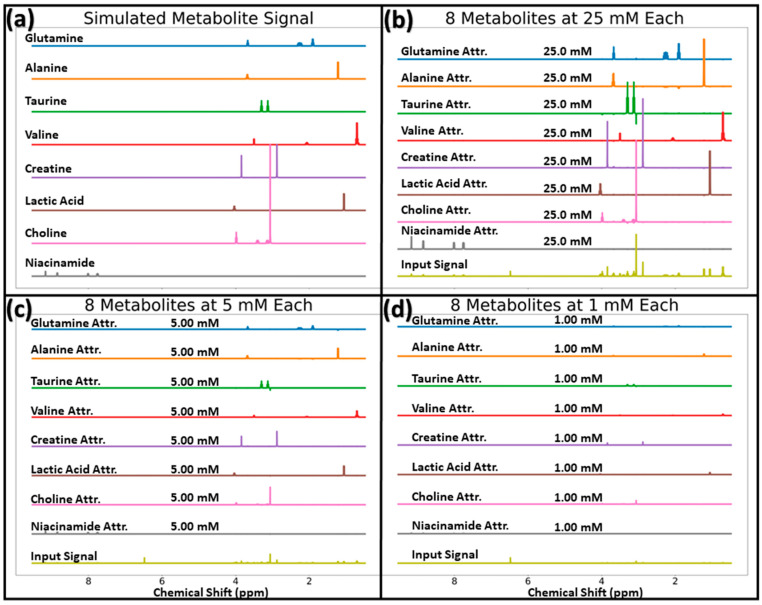
Ground truth metabolite signals (panel (**a**)) for the eight metabolites the model is trained to quantify, and MLP-Sim attribution scores and their respective input signals for inputs with all 8 analytes at 25, 5, and 1 mM for the panels (**b**–**d**), respectively. The predicted concentrations are displayed above each output node’s attribution scores. Abbreviations: Attr. = Attribution scores.

Loss converged for MLP-Sim and MLP-Exp, with the lowest test losses achieved at 2996 (out of 5000) and 16,489 (out of 100,000) epochs, respectively. The IG method was first assessed on three input spectra with all eight analytes present at equal concentrations (25, 5, and 1 mM) using the MLP-Sim network. Figure 3 displays the resulting attribution scores for each of these inputs along with the actual input signal (panels b–d), as well as the ground truth analyte signals for comparison (panel a). This figure notes the model’s high accuracy by showing the predicted concentration for each analyte above its respective attribution scores. Each output node’s attribution scores highly resemble its respective analyte’s ground truth signal in terms of line shape and frequency. Attribution scores scale according to predicted concentration, and the sum of attribution scores for each output node correlates very highly with the predicted concentration (mean absolute percent differences between attribution sum and prediction concentration across all eight metabolites of 0.004%, 0.03%, and 0.14% for mixtures at 25, 5, and 1 mM, respectively). This result confirms that the computed attribution scores accurately reflect the contribution (in the appropriate units, mM) of each input data point to the predicted concentration.

**Figure 4:**
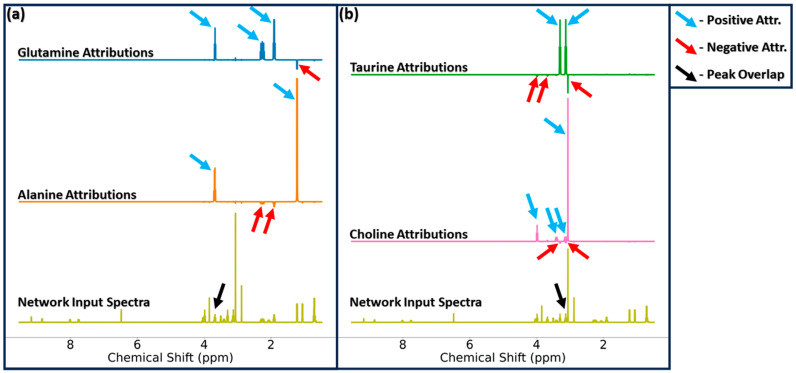
Glutamine and alanine attributions (panel (**a**)) and taurine and choline attributions (panel (**b**)) for the MLP-Sim input spectra of all eight analytes at 25 mM. The black arrow highlights the frequency range in which these signals overlap in the mixture spectrum. Blue arrows denote areas of positive attribution corresponding directly to that output node’s respective metabolite. Red arrows denote frequencies of negative attribution scores which compensate for the increased intensity experienced with the overlapping resonances. Abbreviations: Attr. = Attribution scores.

A closer look at attribution scores reveals a mechanism for dealing with peak overlap. Figure 4 highlights this mechanism for two sets of analytes with overlapping resonances, glutamine/alanine (panel a) and taurine/choline (panel b), for the input of all eight analytes at 25 mM. Glutamine and alanine have peak multiplets which overlap at ~3.7 ppm at 400-MHz, which appears to induce regions of negative attribution at chemical shifts corresponding to the other, non-overlapping signals of the overlapping analyte (i.e., areas of negative attribution for glutamine quantification at frequencies associated with non-overlapping alanine resonances, and areas of negative attribution for alanine quantification at non-overlapping glutamine resonances, all denoted by red arrows in Figure 4). Negative regions of attribution are not seen at frequencies corresponding to the remaining, non-overlapping analytes. The same patterns of positive and negative attribution due to resonance overlap are seen with attributions for taurine and choline which overlap near 3.1 ppm (note that this overlap is seen in the simulated spectra utilized in our study, but experimentally these metabolites will not overlap in this manner).

**Figure 5:**
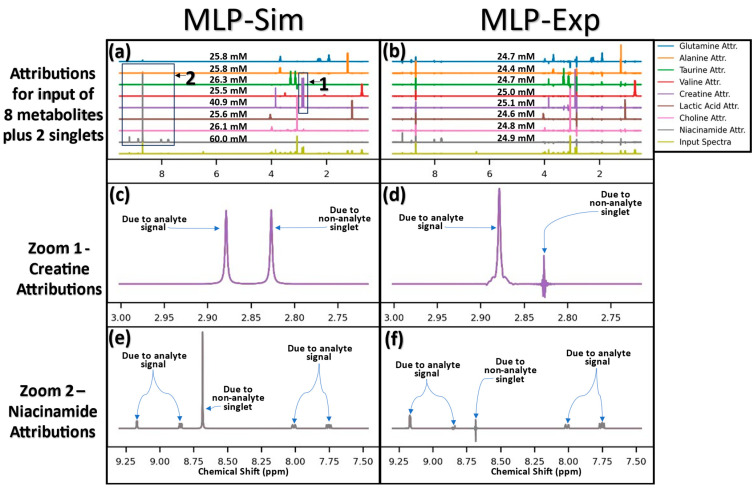
Attributions for MLP-Sim (left side) and MLP-Exp (right side) for the input spectrum of all eight analytes at 25 mM plus two randomly added singlets (peak maxima at ~2.82 and ~8.70 ppm). Zoomed in regions of creatine and niacinamide are shown in the bottom four panels, with the ROIs denoted by “1” and “2” in panel (**a**). The panels show: (**a**) MLP-Sim attribution scores for all 8 metabolites plus the input spectrum, (**b**) MLP-Exp attribution scores for all 8 metabolites plus the input spectrum, (**c**) zoom of MLP-Sim creatine attribution scores, (**d**) zoom of MLP-Exp creatine attribution scores, (**e**) zoom of MLP-Sim niacinamide attribution scores, and (**f**) zoom of MLP-Exp niacinamide attribution scores. Abbreviations: Attr. = attribution scores.

To examine how training dataset modification can affect model behavior, both MLP-Sim and MLP-Exp attribution scores were computed for an input signal of all eight analytes at 25 mM plus two singlets added at random. On the left side of Figure 5, it can be seen that for a model trained in the absence of such interfering signals such as MLP-Sim, the model attributes the non-analyte signals to the nearest analyte. Boxes marked “1” and “2” in panel a are regions of interest shown zoomed-in in panels c and e, respectively. Zoom 1 (panel c) shows a non-analyte singlet signal at ~3.83 ppm wrongly attributed to creatine, and zoom 2 (panel e) shows a non-analyte singlet at ~8.70 ppm wrongly attributed to niacinamide. Panel a includes the predicted concentrations for each metabolite and it is shown that the concentrations for creatine and niacinamide are overestimated while the concentration predictions for the other six metabolites are slightly overestimated. The right side of Figure 5 shows the same scenario for MLP-Exp. Panels d and f are the zoomed-in attribution regions determined with MLP-Exp which show that the non-analyte singlets are interpreted as noisy regions of attribution, and the predicted concentrations are near 25 mM for both creatine and niacinamide. Further, the sums of non-analyte signals in panels c and e are 14.8 and 34.6 mM, respectively, while these regions sum to −0.12 and −0.01 mM for panels d and f, respectively.

As can be seen in Figure 5, the model trained with variations in linewidth, SNR, peak shift, baseline shift, and added non-analyte signals resulted in more complex attributions than seen with MLP-Sim. Unlike MLP-Sim, MLP-Exp attributions at ROIs not directly corresponding to a given node’s analyte show noisy, low-intensity attribution scores (whereas for MLP-Sim these signals were essentially ignored unless they overlapped the target analyte). To better understand these complexities, the attributions for alanine were further examined for the same input of all eight metabolites at 25 mM (Supplementary Figure S2). These ROIs were summed to determine the area of each attribution region (and thus approximate the contribution to predicted concentration of each ROI) and the results are displayed in Supplementary Tables S1 and S2 which, respectively, show each ROI area and the sum of all ROIs per metabolite. ROI areas for non-overlapping metabolites essentially sum to cancel themselves out (e.g., alanine attributions at the four niacinamide resonances have a sum of −0.01). Supplementary Tables S1 and S2 further show ROI sums for an input with six of eight metabolites (leaving out glutamine and taurine, which overlap with alanine and choline, respectively, to observe model behavior without analyte overlap). MLP-Exp attribution scores reveal that overlapping signals induce model behavior similar to the signal overlap compensation seen with MLP-Sim. A further insight from Figure 5 is that not all resonances from a particular analyte are required for quantification as it is shown that only the valine peak near 0.70 ppm has meaningful attribution score intensity. This example is enlarged and compared to ground-truth-simulated analyte signals in Supplementary Figure S3.

### 3.2. XAI with Experimental Lipid Spectra

**Figure 6:**
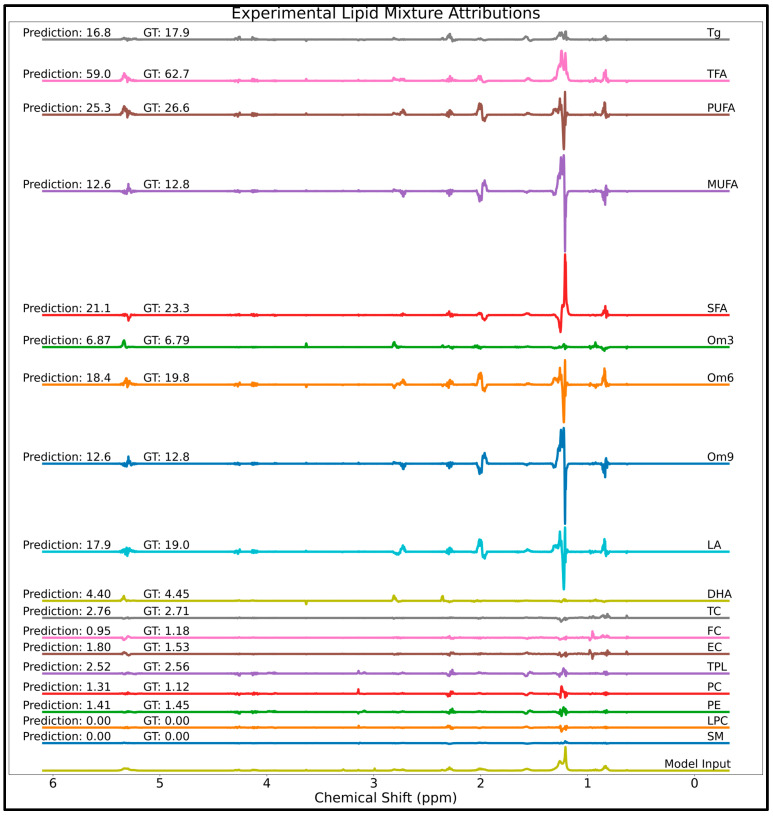
Attributions scores determined on a lipid reference standard mixture spectrum (bottom spectrum) containing tridocosahexaenoin, trilinolein, triolein, tripalmitin, cholesterol, methyl eicosapentaenoate, palmitic acid, cholesteryl linoleate, cholesteryl arachidonate, 1,2-dipalmitoyl-sn-glycero-3-phosphocholine, 1,2-dioleoyl-sn-glycero-3-phosphocholine, 1,2-dipalmitoyl-sn-glycero-3-phosphoethanolamine, 1,2-dimyristoyl-sn-glycero-3-phosphoethanolamine, and SM. Abbreviations: GT = ground truth; DHA = docosahexaenoic acid; EC = esterified cholesterol; FC = free cholesterol; LA = linoleic acid; LPC = lysophosphatidylcholine; MUFA = monounsaturated fatty acids; Om3 = omega-3 fatty acids; Om6 = omega-6 fatty acids; Om9 = omega-9 fatty acids; PC = phosphatidylcholine; PE = phosphatidylethanolamine; PUFA = polyunsaturated fatty acids; SFA = saturated fatty acids; SM = sphingomyelin; TC = total cholesterol; TFA = total fatty acids; Tg = total triglycerides; TPL = total phospholipids.

The XAI approach was next applied to spectra acquired using a 400-MHz NMR spectrometer and an MLP trained for the quantification of lipid groups in mixtures. Attribution scores determined by the IG algorithm for a model input spectrum of a lipid reference standard mixture are shown in Figure 6. Also displayed are the fairly accurate lipid concentrations predicted by the model, as well as ground truth concentrations. As with simulated spectra, the attribution scores determined in the lipid mixture spectra scale with concentration and attributions associated with certain frequencies are positive, negative, or neutral depending on their effect on analyte concentration. To help visualize specific spectral regions, zoomed in regions of the lipid attributions in Figure 6 are supplied as Supplementary Figures S4–S6, with attribution scores scaled to a max intensity of 1.0 for each analyte to help the smaller signals be seen.

The positive regions of attribution for each lipid group largely correspond to resonances expected to contribute to predicted concentrations at a given output node (compare attributions to standard spectra in Figure 2 and to the annotated spectrum denoting important lipid signals provided as Supplementary Figure S1 for help in identifying specific lipid signals and resonances used by spectroscopists). A representative example is discussed for cholesterol attributions (Figure S4). TC shows positive attribution scores from 0.63 to 0.98 ppm corresponding to cholesterol-specific C18, C19, C21, C26, and C27 methyl groups. However, due to the extensive overlap between cholesterol protons and fatty acid (FA) methyl protons (~0.8–0.94 ppm), TC shows negative attribution scores at the (FA) methylene chain (~1.15–1.35 ppm) for compensating for the overlap. The remainder of the TC attribution scores are relatively low amplitude and noisy regions of attribution. For both EC and FC, many signal regions overlap with each other. FC’s largest region of positive attribution is induced by the FC C19 peak at 0.96 ppm, and the attribution quickly transitions into a region of large negative attribution caused by the EC C19 peak at 0.98 ppm to compensate for the extensive overlap with EC. EC reciprocates this behavior, with the model prioritizing the less overlapped peak at 0.98 ppm as the most important for quantification, with a sharp cutoff between the side-by-side overlapping FC and EC resonances, and negative attribution scores to compensate for the overlapped signals of these highly homologous lipid groups.

Many further logical model behaviors are indicated by the attribution scores. The triglyceride-specific glycerol backbone multiplets found near 4.10 and 4.26 ppm are major areas of positive attribution for Tg quantification. The glycerol signals at 4.10 ppm from triglycerides overlap significantly with phospholipid signals, and both Tg and TPL show positive attribution towards their respective analyte and negative attribution towards the overlapping analyte. As expected, olefinic protons near 5.35 ppm show positive attribution scores for FA species containing double bonds like PUFA/Om3/DHA, while very low attribution scores for SFA. In general, the model attributes a significant magnitude of positive concentration with respect to the peak used by spectroscopists for lipid profiling.

**Figure 7:**
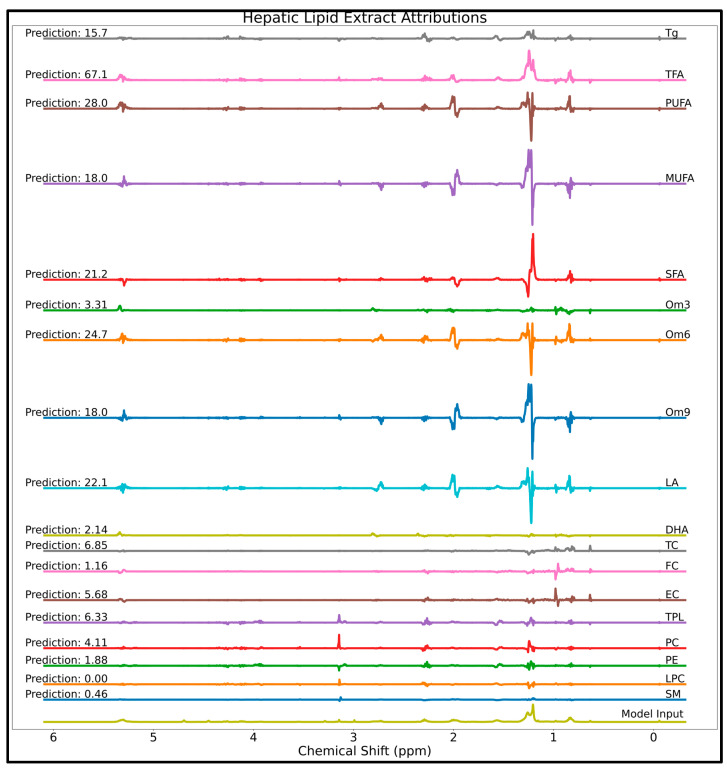
Attributions scores for the 18 lipid analytes determined using the integrated gradients approach on a murine hepatic lipid extract spectrum (bottom spectrum). Abbreviations: DHA = docosahexaenoic acid; EC = esterified cholesterol; FC = free cholesterol; LA = linoleic acid; LPC = lysophosphatidylcholine; MUFA = monounsaturated fatty acids; Om3 = omega-3 fatty acids; Om6 = omega-6 fatty acids; Om9 = omega-9 fatty acids; PC = phosphatidylcholine; PE = phosphatidylethanolamine; PUFA = polyunsaturated fatty acids; SFA = saturated fatty acids; SM = sphingomyelin; TC = total cholesterol; TFA = total fatty acids; Tg = total triglycerides; TPL = total phospholipids.

Attribution scores and predicted concentrations computed on the hepatic extract spectrum are displayed as Figure 7. Zoomed in regions of the lipid attributions in Figure 7 are supplied as Supplementary Figures S7–S9, with attribution scores scaled to a maximum intensity of 1.0 for each analyte. The attribution score patterns discussed above for the lipid standard mixture also apply to the hepatic lipid mixture spectrum. Additionally, a case of bias for the quantification of MUFA and Om9 is seen in the form of nearly identical attributions and thus the same predicted concentration.

## 4. Discussion

This research explored the use of XAI to facilitate the understanding of NN-based quantification of analytes in mixtures measured by NMR spectroscopy. Knowing what influences a model’s predictions can improve user confidence and promote the adoption of NN methods compared to the more conventional, slower, and generally more manual methods that operate via user domain knowledge and/or more widely understood statistical principles. XAI was used to assign scores attributing the magnitude of contribution to quantification for every data point in simulated aqueous metabolite mixture spectra as well as several experimentally acquired lipid spectra. Our results, obtained from simulated and experimental spectra, confirm that the XAI computed attribution scores for each analyte agree accurately with their resonance locations and ground truth concentrations (in the appropriate units, mM).

Using the IG approach promoted an understanding of how NNs make decisions when predicting metabolite concentrations from NMR spectra. Results with simulated aqueous metabolite spectra revealed that, much like a spectroscopist, the models identify and quantify analytes based on their characteristic combination of resonance frequencies, amplitudes, and linewidths. Attribution scores further revealed mechanisms for compensating for peak overlap by subtracting analyte concentration based on the presence and concentration of overlapping analytes. These results were confirmed visually with regions of positive attribution scores highly resembling ground truth simulated signals and regions of negative attribution occurring at frequencies corresponding to competing analyte signals. Summing all attribution scores and specified ROIs for representative inputs provided a quantitative analysis to back up visual results and revealed that attribution scores for a given analyte can be interpreted as the contribution in millimolar of each data point in an input spectrum (a desirable trait for regression XAI, as proposed by Letzgus et al. [10]). Our results are similar to recent XAI regression studies which note that the magnitude of attribution scores reflect the degree of contribution towards a prediction for a given feature, and the sign of attribution scores reflects whether a feature increases or decreases the magnitude of the model output [11,16].

The IG method provides a way to visualize how a model’s behavior can change when utilizing different training datasets. MLP-Sim, which did not see any interfering signals in training, attributed non-overlapping, non-analyte singlets to the analyte with the closest resonance; however, changing to a training dataset implementing randomly inserted and scaled singlets revealed that the network can effectively ignore non-overlapping, non-analyte singlets through noisy regions of attribution scores that integrate to a near-zero magnitude. Comparing MLP-Sim and MLP-Exp attributions revealed that the models take a slightly different approach to metabolite quantification. While MLP-Sim mostly ignored the signals of the seven other analytes for the quantification of each metabolite (except for overlapping resonances), MLP-Exp attributed positive and negative attribution scores to these signals in a manner in which they canceled one another out. Unlike MLP-Sim, MLP-Exp did not always utilize 100% of analyte resonances for the quantification of a given metabolite (e.g., valine).

After an initial validation of the IG method in simulated spectra, the XAI approach was utilized to understand MLP behavior in the task of NMR lipid profiling in proton spectra acquired using a 400-MHz NMR spectrometer. Attribution scores for lipid analytes measured in a complex lipid reference standard mixture and murine hepatic lipid extract revealed similar mechanisms of analyte quantification as seen with simple simulated spectra; however, the larger number of analytes and high structural similarity found among biological lipids contributed to much more extensive overlap and thus more complex attribution allocation by the model. The NN attributes positive concentration to analyte-specific resonances, accounts for overlapping signals, and is generally agnostic towards less significant signals for quantifying a particular analyte. The effectiveness of the model to prioritize the least ambiguous signals for each lipid analyte is further confirmed by noting that each output node attributes a significant level of positive concentration towards peaks used by human spectroscopists for lipid group quantification [5,13,15]. The IG method shows that MLP-based processing for the quantification of analytes in NMR spectra can use the entire spectra for quantification, like line-fitting software (e.g., LipSpin and Chenomx), can focus on individual peaks like a human spectroscopist might, and can flexibly focus on the most important subset of resonances for quantification. The model is able to quantify analytes that do not have any isolated, unambiguous resonances, as seen with lipid groups like TFA, TPL, LPC, Om6, MUFA, and PUFA.

In addition to providing a quantitative means of understanding how a machine learning model makes its decisions, XAI can potentially be used as a tool for detecting bias for model de-bugging. Despite the high degree of homology among lipid structures, the lipid model was trained using only 15 lipid reference standards; therefore, there is potential for bias, especially when scaling up to quantification in tissue extracts which likely contain several orders of magnitude more lipid species. One bias detected in the lipid quantification NN is that MUFA and Om9 quantification have essentially the same set of attribution scores, resulting from the fact that oleic-acid-containing lipids were the only MUFAs or Om9s utilized in model training. This is likely to affect accuracy in the quantification of mixtures containing lipid species the model has not been trained to quantify, such as omega-5 or omega-7 FAs (which are primarily MUFAs), non-oleic acid Om9s, or non-MUFA Om9s. Despite this potential bias, oleic acid is the most abundant and widely distributed FA in nature and thus is expected to be the major Om9 and MUFA in most biological extracts [17,18] and most Om9 are MUFAs [19]; therefore, the effects on the analysis of murine hepatic lipids are likely limited. Training with further types of Om9s and MUFAs should eliminate this bias caused by our limited training dataset.

A second case of bias was noted in the attributions determined in the experimental lipid mixture. TriDHA and mEPA were the only Om3s used in training, with TriDHA being the only DHA-containing compound. This training dataset limitation caused the model to associate the FA methyl ester signal of mEPA with Om3. The model leveraged this FA methyl signal as a way to distinguish between mEPA and the only other omega-3, DHA, causing the node quantifying DHA to associate negative attribution at the FA methyl ester resonance to accommodate peak overlap between these two Om3s (while an unbiased model would not find any association between the FA methyl ester resonance and DHA or Om3). We expect these biases to diminish with increasingly representative training datasets (in this case, adding non-Om3 FA methyl esters and further Om3 species).

The IG gradient method of XAI permitted the user-understanding of what influences MLP-based concentration prediction in analyte mixtures. One limitation of this study is the use of only one XAI method. It is possible that other gradient-based feature attribution methods, or possibly perturbation or contrastive attribution methods, may provide more useful insights, which warrants future investigation. While NN-based quantification can take away much of the burden of user knowledge during metabolite identification and quantification, expertise is required to understand how attribution scores relate to model input spectra. Simulated spectra were used for the aqueous metabolite analysis in this study, and it is worth noting that simulated spectra do not perfectly replicate spectra that are collected experimentally, although they are generally close approximations.

Future work should include testing the current IG method’s effectiveness in NNs more advanced (e.g., convolutional neural networks) than the simple MLP and in multi-dimensional NMR spectra. The NNs used in this study were trained using 8-15 metabolite reference signals, but most biological samples can be expected to have dozens to thousands of metabolites. Future studies should incorporate chemical shift variations inspired by temperature, pH, and other experimental effects and should include more unknown signals (like non-analyte metabolites, randomly placed multiplets, residual lipid/protein signals, and potential contaminants) and more analyte aqueous metabolites to eventually test NN and XAI methods in aqueous tissue extracts. More lipid metabolite signals should be included in training to reduce our current model’s bias, although our XAI approach with the current MLP was still capable of identifying important resonances among the likely thousands of hepatic lipids. The IG method could be used in an exploratory fashion to determine resonances or resonance patterns important for the quantification of an analyte in a given scenario. Further, this XAI approach can be extended to other spectroscopic or similar analyses where users are interested in determining the important portion of a signal for a given task.

## 5. Conclusions

The NN approach has advantages in terms of ease of use, speed, automation, and scalability compared to conventional NMR metabolite profiling methods, and with XAI, concentration prediction need not occur in a black box. The IG algorithm facilitated the understanding that the NN models in this study identify and quantify analytes based on their respective signal frequencies, line-shapes, and amplitudes while prioritizing peaks with less overlap and accounting for resonance overlap with other analytes. This XAI approach allowed us to visualize different model behaviors induced by differing training datasets and to detect potential model biases. Overall, this work confirms the utility of the IG approach to XAI for uncovering which features are most important for analyte quantification in NMR spectra.

## Data Availability

The spectral data and code utilized in this study are openly available on GitHub (https://github.com/tpirneni/XAI-NMR) at https://doi.org/10.5281/zenodo.11639204.

## Supplementary Materials

The following supporting information can be downloaded at: https://www.mdpi.com/article/10.3390/metabo14060332/s1, Figure S1: Annotated structures contributing to lipid resonances; Figure S2: Alanine MLP-Exp attributions; Table S1: Alanine attributions: per-resonance attribution sums; Table S2: Alanine attributions: per-metabolite attribution sums; Figure S3: Valine MLP-Exp attributions; Figure S4: Lipid mixture attributions (0.60–1.38 ppm); Figure S5: Lipid mixture attributions (1.83–3.34 ppm); Figure S6: Lipid mixture attributions (3.48–5.40 ppm); Figure S7: Hepatic lipid extract attributions (0.60–1.38 ppm); Figure S8: Hepatic lipid extract attributions (1.83–3.34 ppm); Figure S9: Hepatic lipid extract attributions (3.48–5.40 ppm).
