# Supplementary material for: Explainable AI to Facilitate Understanding of Neural Network-Based Metabolite Profiling Using NMR Spectroscopy
Source: Metabolites. 2024 Jun 14;14(6):332. doi: 10.3390/metabo14060332 (PMC11205398; doi:10.3390/metabo14060332)
Supplement: Supplementary file 1 [file metabolites-14-00332-s001.zip › metabolites-3045535-supplementary.pdf]

Supplementary Information

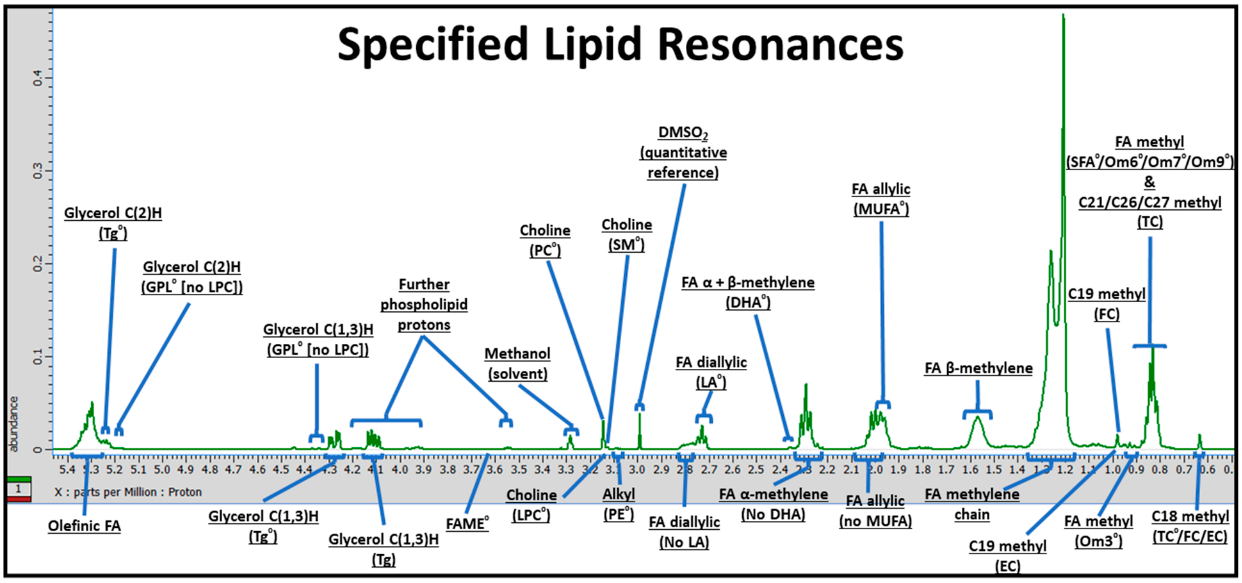

Figure S1. – Annotated Structures Contributing to Lipid Resonances. Signal regions for ^{1}H-NMR profiling of common biological lipid groups (figure adapted from "LipSpin: A New Bioinformatics Tool for Quantitative 1H NMR Lipid Profiling" by Gil et al). The molecular structure is specified for each of the denoted regions, and in parenthesis is mentioned the most exclusive lipid grouping quantifiable at this resonance or other resonance specific information. The '^{0}' symbol denotes resonances generally used by spectroscopists for quantification of a specified lipid group. Om7 fatty acids weren't used to train our NN; although, they are highly likely to be present in our hepatic extract. Abbreviations: FA = fatty acids; FPL = glycerophospholipids; DHA = docosahexaenoic acid; EC = esterified cholesterol; FC = free cholesterol; LA = linoleic acid; LPC = lysophosphatidylcholine; MUFA = monounsaturated fatty acids; Om3 = omega-3 fatty acids; Om6 = omega-6 fatty acids; Om7 = omega-7 fatty acids; Om9 = omega-9 fatty acids; PC = phosphatidylcholine; PE = phosphatidylethanolamine; SFA = saturated fatty acids; SM = sphingomyelin; TC = total cholesterol; Tg = total triglycerides; FAME = fatty acid methyl ester.

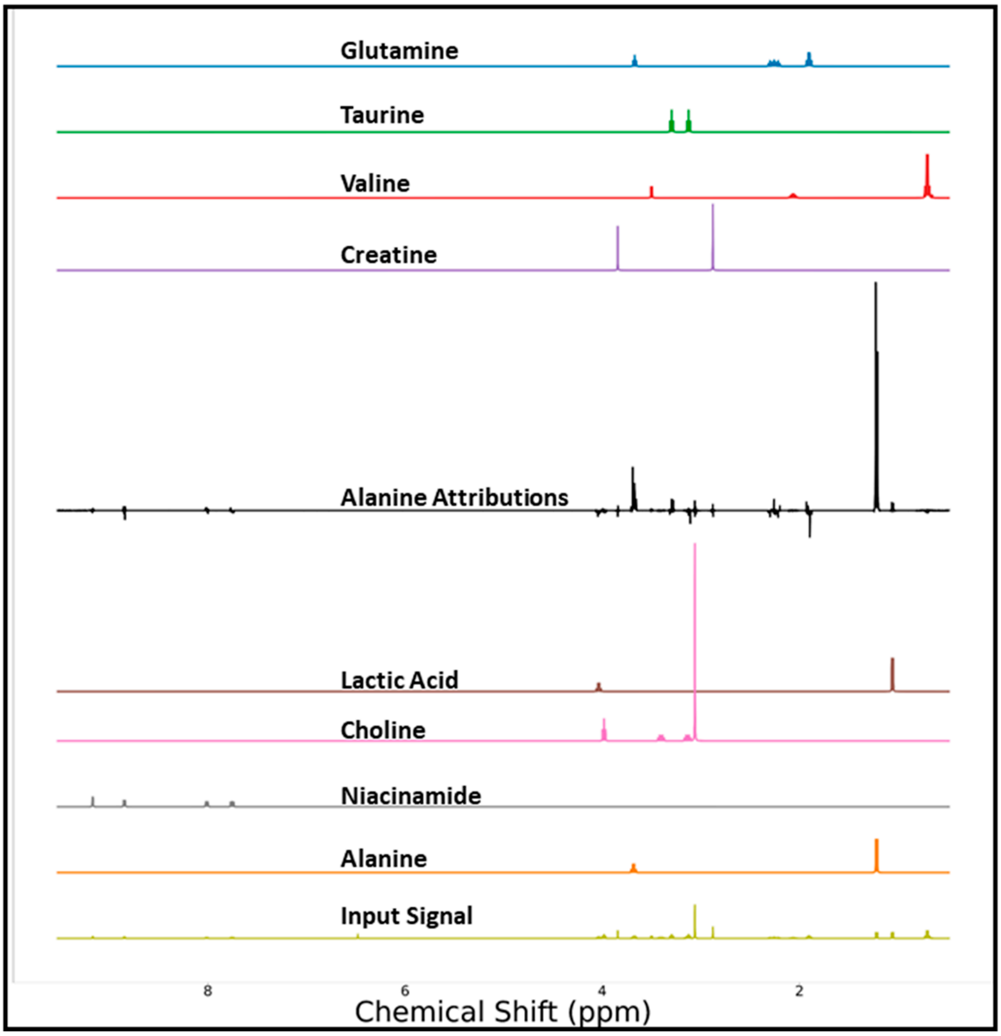

Figure S2. – Alanine MLP-Exp Attributions. MLP-Exp attribution scores for alanine (middle black signal) and their relationships to ground truth analyte signals (surrounding 8 spectra) at the same chemical shift. The bottom signal is the actual network input of all 8 analytes at 25 mM. Regions of attribution for each multiplet and metabolite are summed in Supplemental Tables S1 and S2, respectively, to highlight the contribution of each resonance to quantification of alanine. Abbreviations: Attr. = attribution scores, ppm = parts per million.

Attribution scores for alanine were examined for a model input of all eight metabolites at 25 mM as seen in Figure S2. These ROIs were summed to determine the area of each attribution region (and thus approximate the contribution to predicted concentration of each ROI) and the results are displayed in Supplementary Tables S1 and S2 which respectively show each ROIs area and the sum all ROIs per metabolite. ROI areas for non-overlapping metabolites essentially work to cancel themselves out. For example, the four ROIs associated with niacinamide sum to -0.24, 0.20, -0.02, and 0.05 at 7.76, 8.02, 8.85, and 9.17 ppm respectively, and the overall sum of these four ROIs is -0.01 leading to an overall negligible effect on alanine quantification. This pattern remains for all non-overlapping analytes.

MLP-Exp attribution scores reveal that overlapping signals induce a model behavior similar to the signal overlap compensation seen with MLP-Sim. Given the complexity of the overlapping signal regions, an additional input was analyzed for comparison which had no overlapping analytes (due to leaving out glutamine and taurine - the other six metabolites were present at 25 mM), and values for summed attribution scores of ROIs and metabolites are shown in Supplementary Table S1 and S2, respectively. MLP-Exp recognizes the alanine signal at 1.24 ppm as highly important in both inputs with and without overlapping metabolites, and in both cases this region contributes around 22.2 mM worth of alanine concentration. When no overlap is present, MLP-Exp attributes the

remaining ~2.9 mM to the other alanine signal at 3.67 ppm. In the presence of overlap, the additional intensity of glutamine near 3.67 ppm causes this ROI's attribution sum to rise to 5.0 mM. Like MLP-Sim, the remaining, non-overlapping glutamine resonances contribute negative attribution at magnitudes of -1.50 and -0.66 mM at 1.90 and 2.26 ppm, respectively (while in the absence of glutamine they contribute only -0.0004 and -0.0003 mM to alanine con-centration). Similarly, with taurine and choline it is shown quantitatively that taurine and choline compensate one another's overlap in the case of overlap but not if no overlap is present (ROI sums in Supplementary Table S1 and S2). Not all resonances from a particular analyte are required for quantification as shown in Supplementary Figure S3 that only the valine peak near 0.70 ppm has meaningful attribution score intensity.

Table S1. - Alanine Attributions: Per Resonance Attribution Sums. Sum of alanine attribution scores at specified ROIs corresponding to target analyte and non-target analyte signals. Abbreviations: ROIs = regions of interest.

**Contributing Metabolite**	**Approximate Chemical Shift (ppm)**	**ROI Attribution Sums - All 8 Metabolites**	**ROI Attribution Sums - No Glutamine/Taurine (6 Metabolites)**
Valine	0.70	−0.06	−0.05
Lactic Acid	1.06	0.32	0.32
Alanine	1.24	22.20	22.19
Glutamine	1.90	−1.50	−0.0004
Valine	2.07	0.01	0.002
Glutamine	2.26	−0.66	−0.0003
Creatine	2.88	0.04	0.03
Choline	3.06	0.25	0.24
Taurine/Choline	3.15	−0.75	−0.08
Taurine	3.30	0.65	0.004
Choline	3.39	−0.02	−0.02
Valine	3.49	0.02	0.02
Glutamine/Alanine	3.67	5.03	2.88
Creatine	3.84	−0.05	−0.04
Choline	3.98	−0.15	−0.15
Lactic Acid	4.05	−0.36	−0.36
Niacinamide	7.76	−0.24	−0.24
Niacinamide	8.02	0.20	0.20
Niacinamide	8.85	−0.02	−0.03
Niacinamide	9.17	0.05	0.06

Table S2. – Alanine Attributions: Per Metabolite Attribution Sums. Sum of alanine attribution scores at specified ROIs corresponding to target metabolite and non-target metabolites signals. Abbreviations: ROIs = regions of interest, Attr. = attributions scores.

**Contributing Metabolite**	**Per Metabolite Attr. Sums - All 8 Metabolites**	**Per Metabolite Attr. Sums - No Glutamine/Taurine (6 Metabolites)**
Alanine + Glutamine	−0.02	−0.01
Taurine + Choline	25.07	25.08
Creatine	−0.02	−0.02
Lactic Acid	−0.04	−0.04
Valine	−0.03	−0.03
Niacinamide	−0.01	−0.01
All Together	24.96	24.97

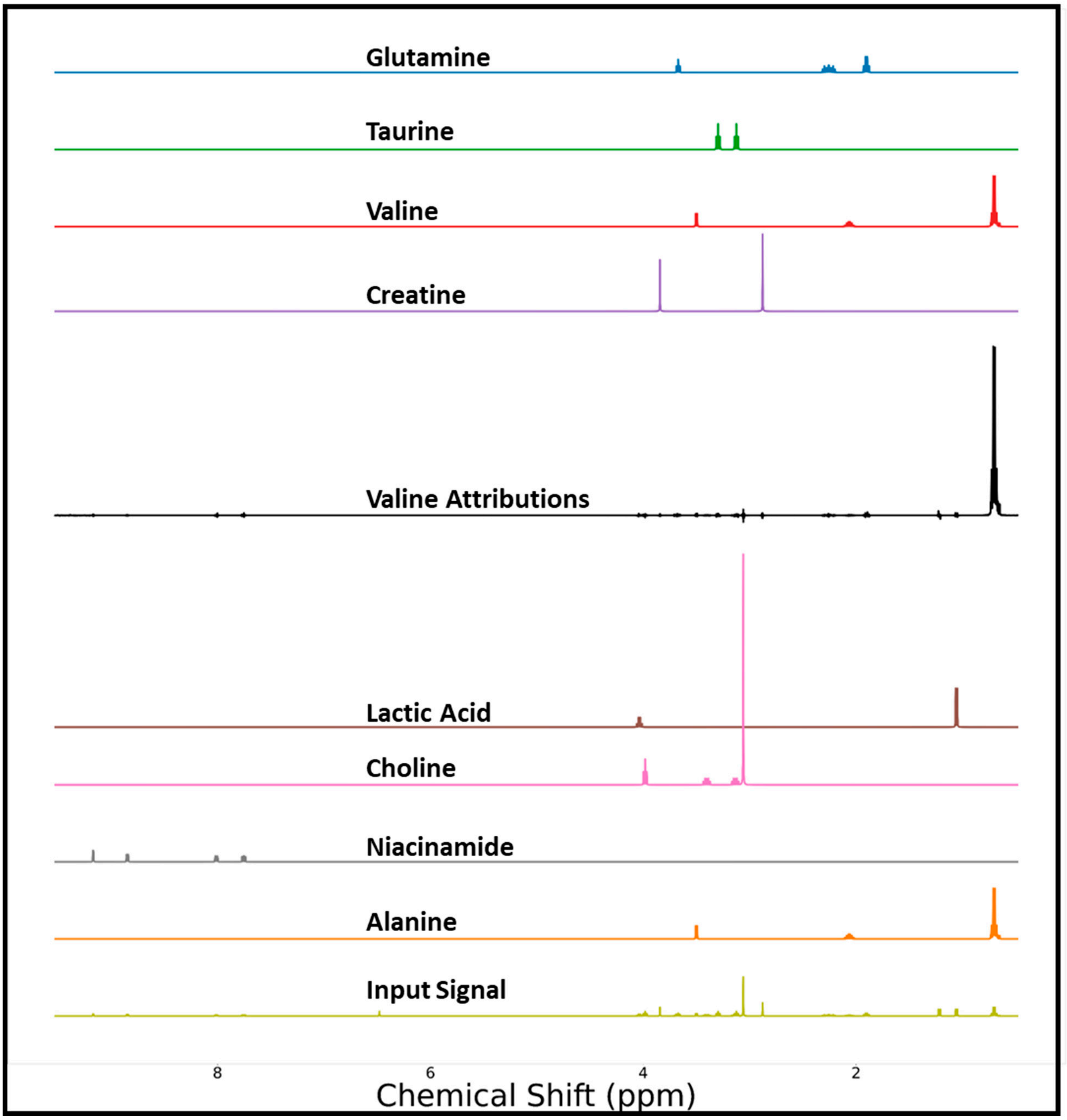

Figure S3. – Valine MLP-Exp Attributions. MLP-Exp attribution scores for valine (middle black signal) and their relationships to ground truth analyte signals (surrounding 8 spectra) at the same chemical shift. The bottom signal is the network input of all 8 analytes at 25 mM. Abbreviations: Attr. = attribution scores, ppm = parts per million.

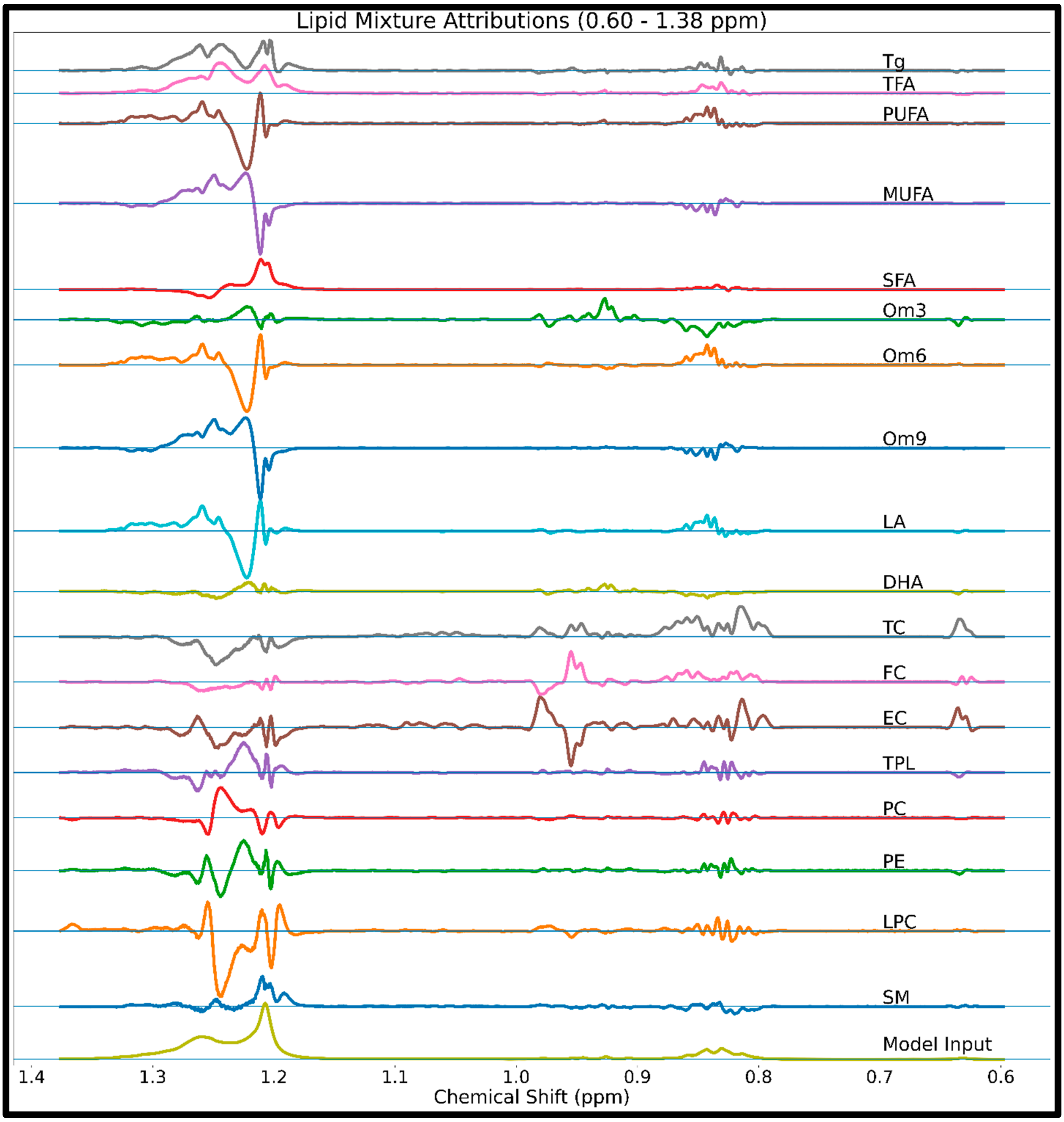

Figure S4. – Lipid Mixture Attributions (0.60 – 1.38 ppm). Attribution scores for lipid groups in the 0.60 through 1.38 ppm spectral region quantified in a mixture of lipid reference standards. Attribution scores are normalized to a maximum signal intensity of 1. The bottom spectrum is the actual model input. Abbreviations: ppm = parts per million; DHA = docosahexaenoic acid; EC = esterified cholesterol; FC = free cholesterol; LA = linoleic acid; LPC = lysophosphatidylcholine; MUFA = monounsaturated fatty acids; Om3 = omega-3 fatty acids; Om6 = omega-6 fatty acids; Om9 = omega-9 fatty acids; PC = phosphatidylcholine; PE = phosphatidylethanolamine; PUFA = polyunsaturated fatty acids; SFA = saturated fatty acids; SM = sphingomyelin; TC = total cholesterol; TFA = total fatty acids; Tg = total triglycerides; TPL = total phospholipids.

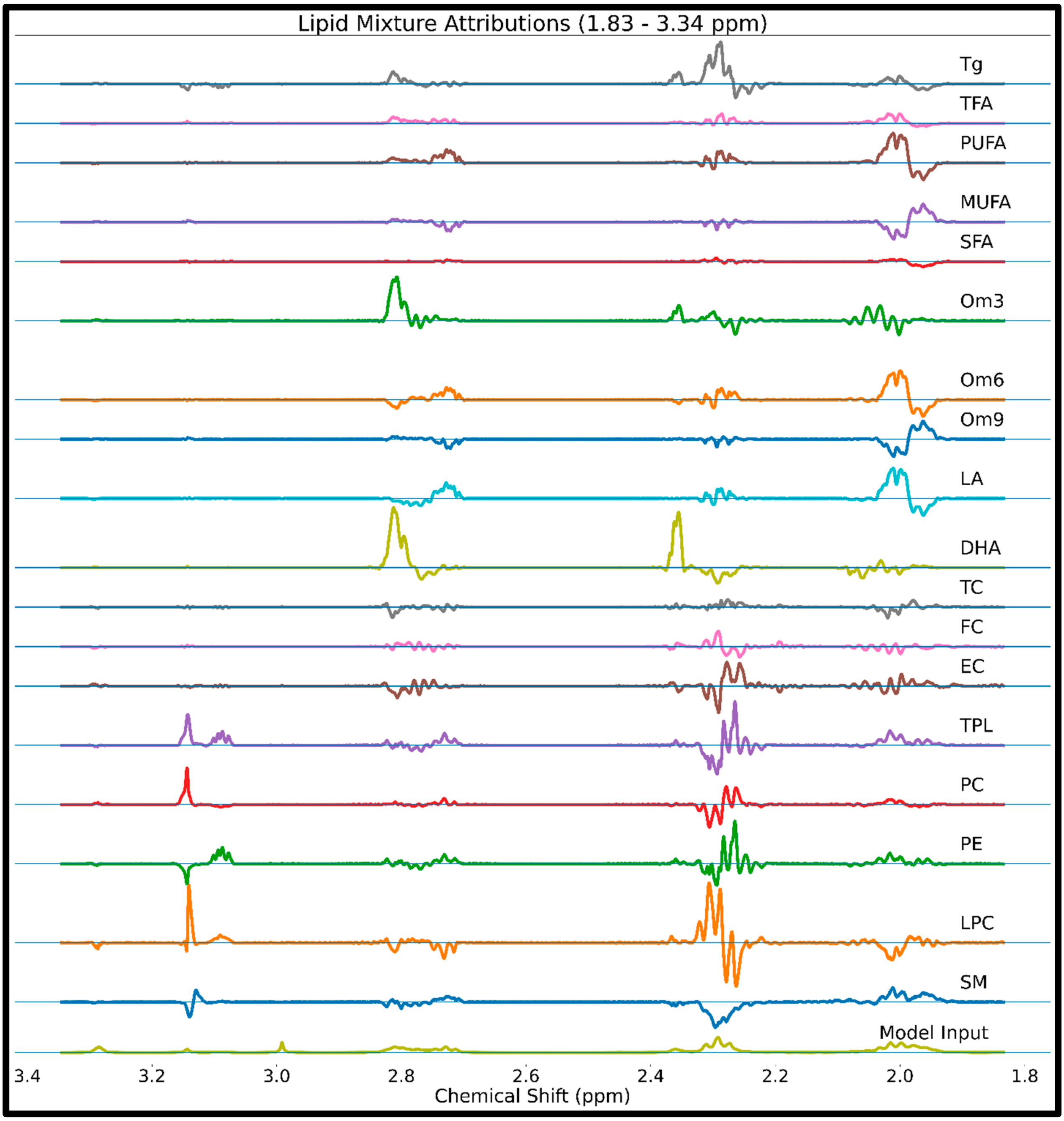

Figure S5. – Lipid Mixture Attributions (1.83 – 3.34 ppm). Attribution scores for lipid groups in the 1.83 through 3.34 ppm spectral region quantified in a mixture of lipid reference standards. Attribution scores are normalized to a maximum signal intensity of 1. The bottom spectrum is the actual model input. Abbreviations: ppm = parts per million; DHA = docosahexaenoic acid; EC = esterified cholesterol; FC = free cholesterol; LA = linoleic acid; LPC = lysophosphatidylcholine; MUFA = monounsaturated fatty acids; Om3 = omega-3 fatty acids; Om6 = omega-6 fatty acids; Om9 = omega-9 fatty acids; PC = phosphatidylcholine; PE = phosphatidylethanolamine; PUFA = polyunsaturated fatty acids; SFA = saturated fatty acids; SM = sphingomyelin; TC = total cholesterol; TFA = total fatty acids; Tg = total triglycerides; TPL = total phospholipids.

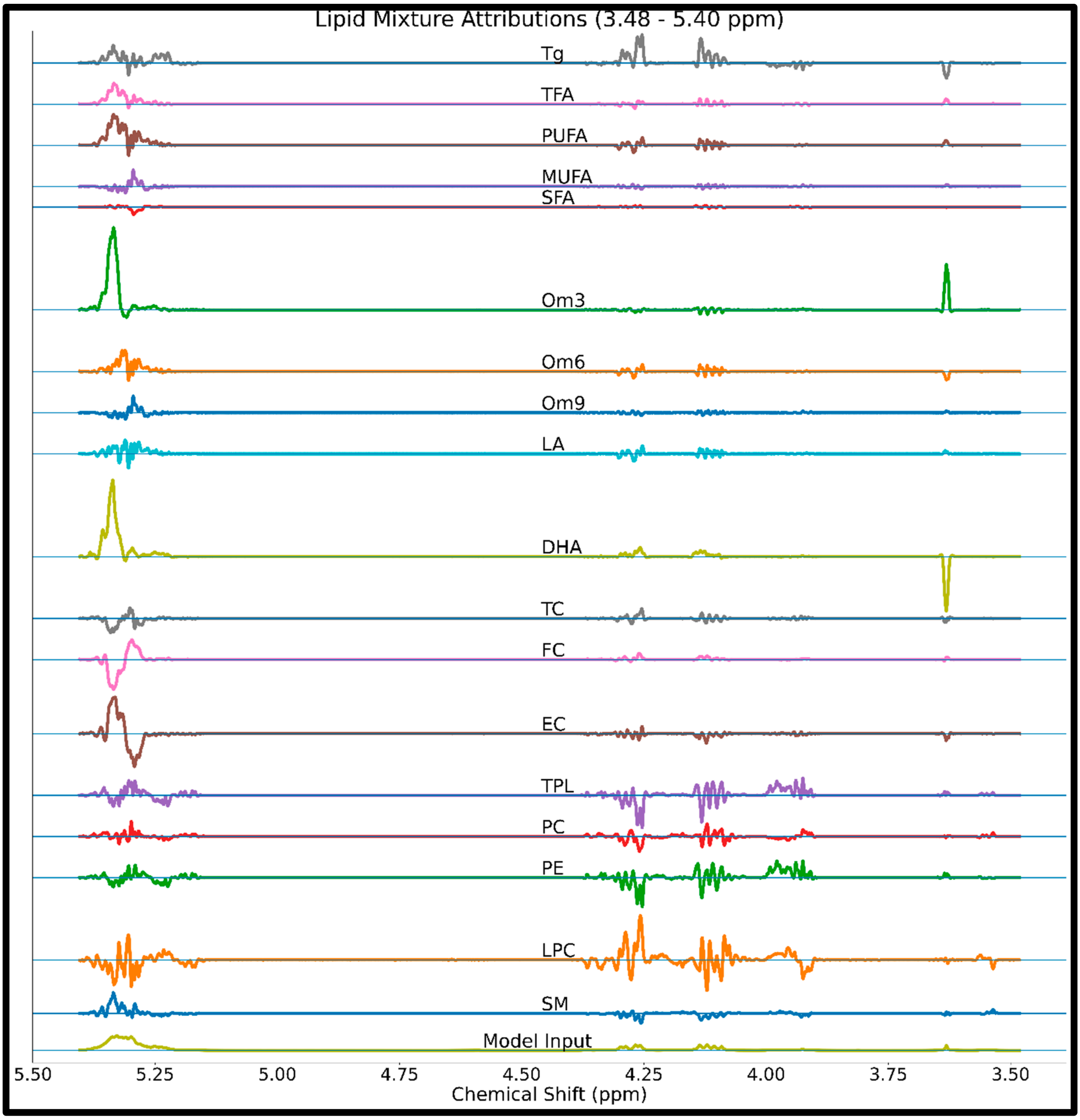

Figure S6. – Lipid Mixture Attributions (3.48 – 5.40 ppm). Attribution scores for the 3.48 through 5.40 ppm spectral region quantified in a mixture of lipid reference standards. Attribution scores are normalized to a maximum signal intensity of 1. The bottom spectrum is the actual model input. Abbreviations: ppm = parts per million; DHA = docosahexaenoic acid; EC = esterified cholesterol; FC = free cholesterol; LA = linoleic acid; LPC = lysophosphatidylcholine; MUFA = monounsaturated fatty acids; Om3 = omega-3 fatty acids; Om6 = omega-6 fatty acids; Om9 = omega-9 fatty acids; PC = phosphatidylcholine; PE = phosphatidylethanolamine; PUFA = polyunsaturated fatty acids; SFA = saturated fatty acids; SM = sphingomyelin; TC = total cholesterol; TFA = total fatty acids; Tg = total triglycerides; TPL = total phospholipids.

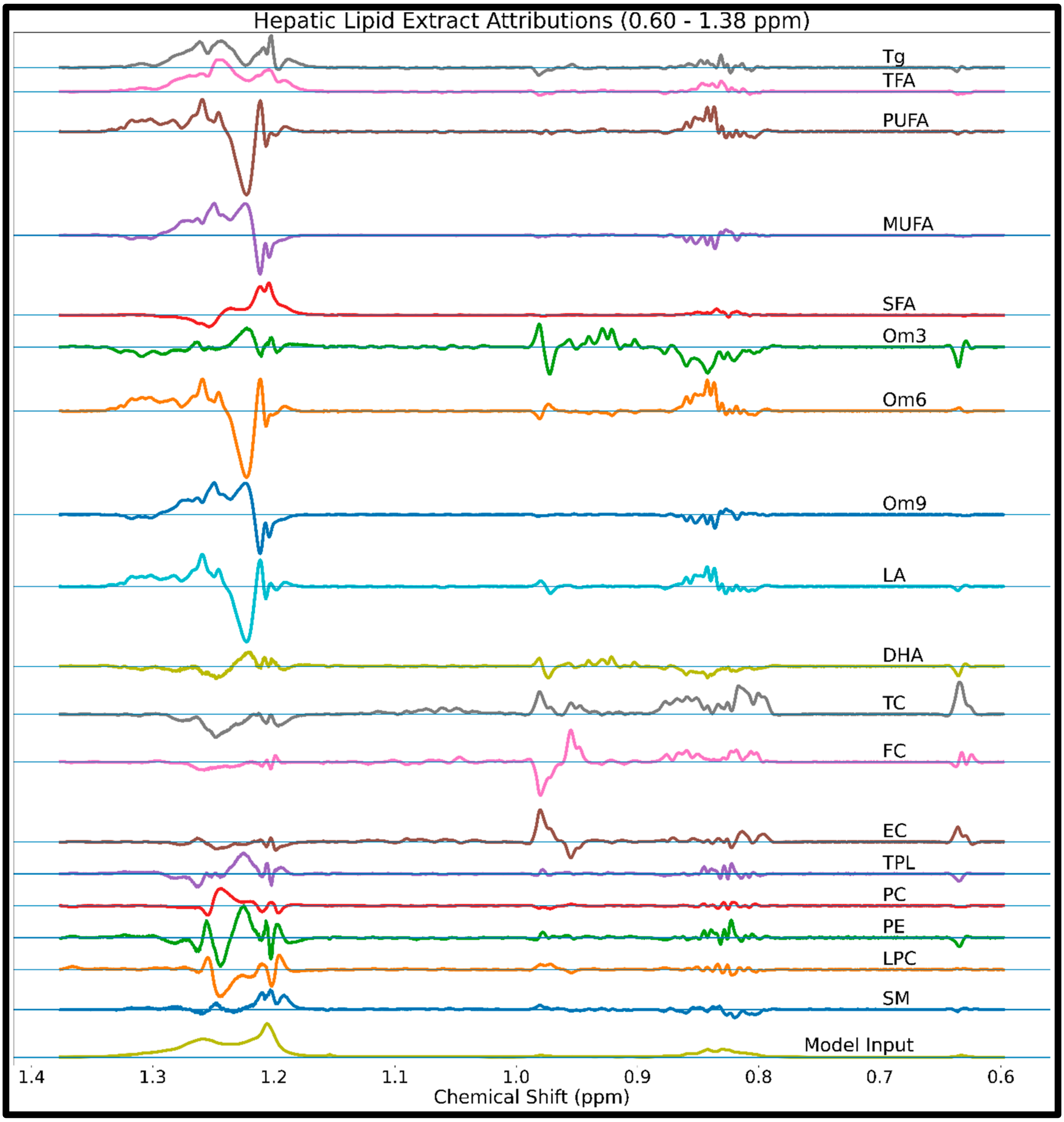

Figure S7. – Hepatic Lipid Extract Attributions (0.60 – 1.38 ppm). Attribution scores for lipid groups in the 0.60 through 1.38 ppm spectral region quantified in a murine lipophilic hepatic extract. Attribution scores are normalized to a maximum signal intensity of 1. The bottom spectrum is the actual model input. Abbreviations: ppm = parts per million; DHA = docosahexaenoic acid; EC = esterified cholesterol; FC = free cholesterol; LA = linoleic acid; LPC = lysophosphatidylcholine; MUFA = monounsaturated fatty acids; Om3 = omega-3 fatty acids; Om6 = omega-6 fatty acids; Om9 = omega-9 fatty acids; PC = phosphatidylcholine; PE = phosphatidylethanolamine; PUFA = polyunsaturated fatty acids; SFA = saturated fatty acids; SM = sphingomyelin; TC = total cholesterol; TFA = total fatty acids; Tg = total triglycerides; TPL = total phospholipids.

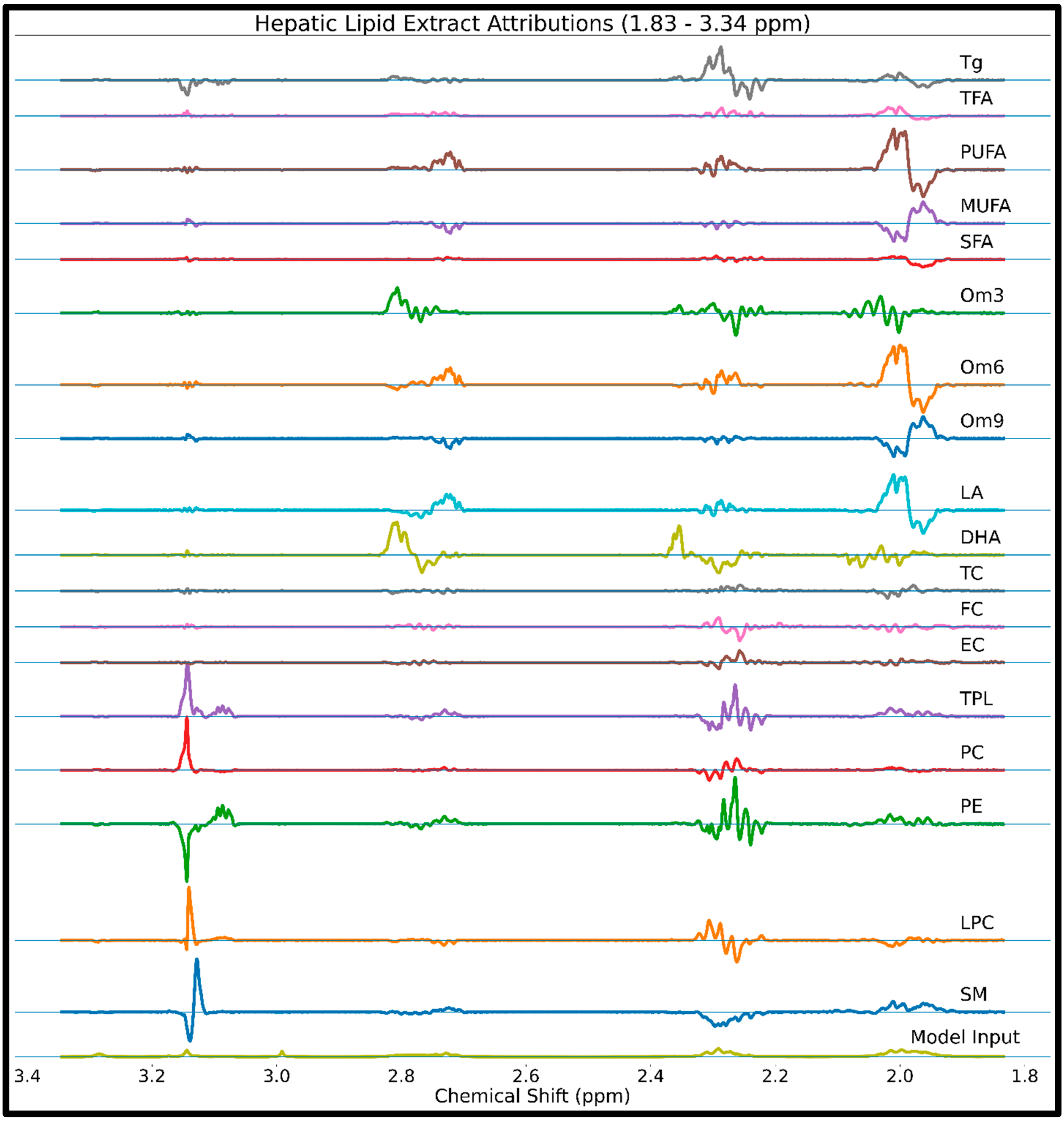

Figure S8. – Hepatic Lipid Extract Attributions (1.83 – 3.34 ppm). Attribution scores for lipid groups in the 1.83 through 3.34 ppm spectral region quantified in a murine lipophilic hepatic extract. Attribution scores are normalized to a maximum signal intensity of 1. The bottom spectrum is the actual model input. Abbreviations: ppm = parts per million; DHA = docosahexaenoic acid; EC = esterified cholesterol; FC = free cholesterol; LA = linoleic acid; LPC = lysophosphatidylcholine; MUFA = monounsaturated fatty acids; Om3 = omega-3 fatty acids; Om6 = omega-6 fatty acids; Om9 = omega-9 fatty acids; PC = phosphatidylcholine; PE = phosphatidylethanolamine; PUFA = polyunsaturated fatty acids; SFA = saturated fatty acids; SM = sphingomyelin; TC = total cholesterol; TFA = total fatty acids; Tg = total triglycerides; TPL = total phospholipids.

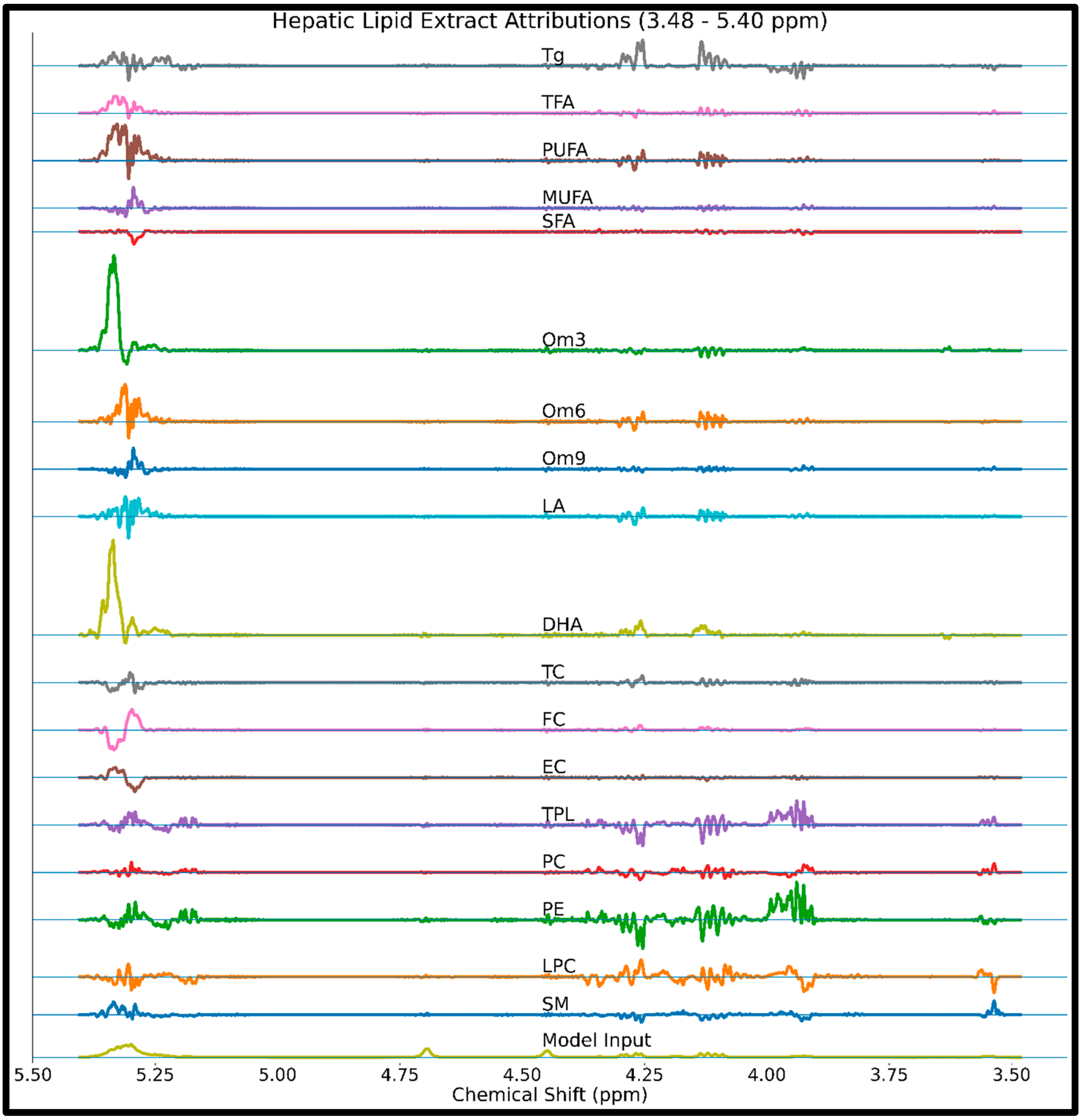

Figure S9. – Hepatic Lipid Extract Attributions (3.48 – 5.40 ppm). Attribution scores for the 3.48 through 5.40 ppm spectral region quantified in a murine lipophilic hepatic extract. Attribution scores are normalized to a maximum signal intensity of 1. The bottom spectrum is the actual model input. Abbreviations: ppm = parts per million; DHA = docosahexaenoic acid; EC = esterified cholesterol; FC = free cholesterol; LA = linoleic acid; LPC = lysophosphatidylcholine; MUFA = monounsaturated fatty acids; Om3 = omega-3 fatty acids; Om6 = omega-6 fatty acids; Om9 = omega-9 fatty acids; PC = phosphatidylcholine; PE = phosphatidylethanolamine; PUFA = polyunsaturated fatty acids; SFA = saturated fatty acids; SM = sphingomyelin; TC = total cholesterol; TFA = total fatty acids; Tg = total triglycerides; TPL = total phospholipids.
